# Translation and cultural adaptation of the *Positive Aspects of Caregiving* Scale for caregivers of people living with dementia in Brazilian context: a methodological study

**DOI:** 10.1590/1516-3180.2023.0325.R1.23012024

**Published:** 2024-03-18

**Authors:** Sofia Cristina Iost Pavarini, Aline Cristina Martins Gratão, Camila Rafael Ferreira Campos, Diana Quirino Monteiro, Elizabeth Joan Barham, Fabiana de Souza Orlandi, Gabriela Martins, Gustavo Carrijo Barbosa, Keila Cristianne Trindade da Cruz, Larissa Corrêa, Luana Aparecida da Rocha, Ludmyla Caroline de Souza Alves, Ana Carolina Ottaviani

**Affiliations:** I PhD. Nurse, Associate Professor, Department of Gerontology, Universidade Federal de São Carlos (UFSCAR), São Carlos (SP), Brazil.; II PhD. Associate Professor, Department of Gerontology, Universidade Federal de São Carlos, São Carlos (SP), Brazil.; III PhD. Psychologist, Postdoctoral Fellow, Postgraduate Program in Psychology, Universidade Federal de São Carlos (UFSCAR), São Carlos (SP), Brazil.; IV PhD. Gerontologist, Postdoctoral Fellow, Department of Gerontology, Universidade Federal de São Carlos (UFSCAR), São Carlos (SP), Brazil.; V PhD. Psychologist, Associate Professor, Department of Psychology, Universidade Federal de São Carlos (UFSCAR), São Carlos (SP), Brazil.; VI PhD. Nurse, Associate Professor, Department of Gerontology, Universidade Federal de São Carlos (UFSCAR), São Carlos (SP), Brazil.; VII MSc. Gerontologist, PhD student, Postgraduate Program in Nursing, Universidade Federal de São Carlos (UFSCAR), São Carlos (SP), Brazil.; VIII MSc. Physiotherapist, PhD student, Postgraduate Program in Nursing, Universidade Federal de São Carlos (UFSCAR), São Carlos (SP), Brazil.; IX PhD. Nurse, Associate professor, Department of Nursing, Universidade de Brasília (UnB), Brasília (DF), Brazil.; X MSc. Gerontologist, PhD student, Postgraduate Program in Nursing, Universidade Federal de São Carlos (UFSCAR), São Carlos (SP), Brazil.; XI MSc. Gerontologist, PhD student, Postgraduate Program in Nursing, Universiade Federal de São Carlos (UFSCAR), São Carlos (SP), Brazil.; XII MSc. Gerontologist, PhD student, Postgraduate Program in Nursing, Universidade Federal de São Carlos (UFSCAR), São Carlos (SP), Brazil.; XIII PhD. Gerontologist, Postdoctoral Fellow, Department of Gerontology, Universidade Federal de São Carlos (UFSCAR), São Carlos (SP), Brazil.

**Keywords:** Dementia, Caregivers, Transcultural study, Mental health, Caregivers, Positive aspects of caregiving, Adaptation of instruments, Major neurocognitive disorder, Family caregiver

## Abstract

**BACKGROUND:**

The Positive Aspects of Caregiving (PAC) scale is used to assess psychosocial benefits provided to caregivers by the task of caring. The PAC scale consists of nine items, assessed using a five-point Likert scale, with higher values indicating greater positive perceptions and gains from the caregiving experience.

**OBJECTIVE:**

To translate and culturally adapt the PAC scale for informal Brazilian caregivers of people with dementia.

**DESIGN AND SETTING:**

A methodological study was conducted at the Federal University of São Carlos.

**METHODS:**

The following stages were carried out: Translation; Synthesis of the translations; Back-translation; Evaluation by an experts’ committee; and Pre-test.

**RESULTS:**

Two independent professionals translated the PAC scale. The consensus version was obtained by merging both translations, which were back-translated into English by a third translator. The expert committee comprised three specialists in the area and project researchers. All scale items presented a Content Validity Index of 1 (CVI = 1.0), and thus remained in the pre-final version of the instrument. The instrument was pre-tested with seven caregivers of people with dementia, the majority of whom were women (57.1%), with a degree of kinship corresponding to sons/daughters (57.1%) and an average age of 55.2 (± 4.1) years. The caregivers considered it clear and understandable and made no suggestions for changes.

**CONCLUSION:**

The PAC scale was translated and culturally adapted for use by informal caregivers of people with dementia in Brazil. However, a psychometric analysis of the instrument is necessary to provide normative data for this population group.

## INTRODUCTION

The Positive Aspects of Caregiving (PAC) can be defined as caregivers’ gains or satisfaction resulting from the care experience and comprise different dimensions, such as perception of gain or reward, satisfaction, resilience, self-efficacy, self-esteem, sense of mastery, personal growth, and sense of life purpose.^1^ ^,^ ^2^ ^,^ ^3^

The PAC scale is widely used to assess the psychosocial benefits provided to caregivers by care tasks. The PAC scale was developed in 2004 by Tarlow et al. and consists of nine items that present assertions about a caregiver’s mental or affective state associated with the care experience. It consists of a five-point Likert scale from (1) disagree a lot to (5) agree a lot, with total scores varying from 9 to 45 points and higher values indicating greater positive perceptions and gains from the caregiving experience.^4^

The PAC scale comprises two factors: Self-affirmation and Life perspective. The Self-affirmation factor, comprising six items, describes the confident and capable self-image of the caregiver. The Life perspective factor, which includes three items, describes improved interpersonal relationships and positive views of life. Internal consistency was α = 0.89 for the overall instrument.^4^

A systematic review of 53 studies aimed at exploring how the PAC affect the well-being of caregivers of people with dementia verified that they are associated with better mental health and quality of life, satisfaction with life, competence, and self-efficacy, as well as with lower levels of depressive symptoms and burden.^2^ A longitudinal study which included 1,283 informal caregivers of people with dementia identified associations between the PAC and caregivers’ well-being and satisfaction with life.^5^

Informal caregivers frequently face stressful situations, including high task demands, physical wear out, financial problems, social isolation, and free time restrictions.^6^ ^,^ ^7^ Previous studies have shown that caring for a person with dementia is associated with higher burden levels, more severe depressive symptoms, and psychological stress in caregivers.^7^ ^,^ ^8^ ^,^ ^9^

Studies on how the PAC scale is used have already been conducted with caregivers of people with dementia in Portugal,^10^ the United States,^11^ Greece,^12^ Singapore,^13^ and Japan.^14^ In Brazil, the PAC are still less explored, and there is no translated and adapted version of the scale for the Brazilian context. Therefore, having a Brazilian version of the PAC scale is relevant, especially for informal caregivers of people with dementia.

## OBJECTIVE

This study aimed to translate and culturally adapt the PAC scale for the informal caregivers of people with dementia in the Brazilian context.

## METHODS

**Figure 1.**
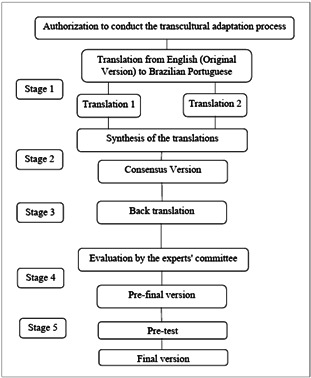
Representation of the methodological model and stages followed in the translation and transcultural adaptation process corresponding to the positive aspects of caregiving scale.

This was a methodological study for the translation and transcultural adaptation of the PAC scale from English to Brazilian Portuguese. The adaptation process followed the stages recommended by Beaton et al.^15^ to achieve semantic, idiomatic, cultural, and conceptual equivalence between the original instrument and the adapted version. Figure **1** illustrates the stages required to transculturally adapt an instrument.

Following the methodological modeling steps, authorization was requested from the author of the original scale to create a new Brazilian version. The researchers rigorously followed all scientific and ethical guidelines and the research was approved by the Committee of Ethics in Research with Human Beings of the Federal University of São Carlos (CAAE No. 88157118.0.1001.5504/ April 05, 2022).

In the first stage, the original version (written in American English) was translated by two qualified and independent translators, one from the health field and the other from psychology, and both proficient in English. The translation process undertaken by the two translators allowed for the detection of errors derived from divergent interpretations of ambiguous terms in the original language. The translations were produced by the researchers and translators working as teams in the second stage, thus creating a consensus version of the PAC scale. The subsequent back-translation stage involved a third translator who was fluent in both languages and native English speaker; however, this person was unaware of the objective of the current study. The instrument was sent to the authors for analysis and approval.

In the fourth stage, evaluation by the experts’ committee, the specialists reviewed and compared all the translations produced with the objective of reaching a modified and adapted version for use in Brazil. The committee consisted of three judges fluent in the original language of the scale, with PhDs in academic training in Health and Psychology and experience with informal caregivers of people with dementia, and project researchers involved in the process. The Content Validity Index (CVI) was used for data analysis, employing a four-point Likert scale, where items scored as 1 and/or 2 were reviewed or excluded, and those scored as 3 and/or 4 were part of the calculation. An agreement value was calculated based on adding up each of the judges’ answers to each item divided by the total number of answers, with a minimum recommended result of 0.78 to confirm equivalence of the instrument after the entire process.^16^ After this evaluation, the pre-final version of the PAC scale was obtained.

In the fifth and last stages, the PAC scale was pre-tested in a convenience sample using the data saturation technique. In total, the sample comprised seven family caregivers of people with dementia with different profiles (sex, age, and degree of kinship), divided into two groups: four caregivers at the first moment and three caregivers later. In this study, we decided to use this test technique by administering the instrument in two groups. The objective of dividing the participants was to gather notes from the first group and improve the writing of the items for better understanding by the second group. Before fully answering the questionnaire, the participants agreed to participate in the study by signing the Free and Informed Consent Form. The pre-test stage was conducted from July to September 2022 in an online format using *Google Forms*. The participants were asked about difficulties in filling the questionnaire or understanding the purpose or meaning of the questions. After the interviews, the experts’ committee discussed the results and proposed the final version.

## RESULTS

Two versions were obtained, which were translated independently: T1 and T2. Once the translations were produced, the researchers met the translators to analyze and evaluate any and all discrepancies between both translated versions (T1 and T2), in addition to comparing them to the original instrument. Both translations were compared to reach consensus. The T1 and T2 versions were considerably similar. There were no significant translation difficulties; however, in the items that presented discrepancies, the translation that the study researchers considered to better express the original meaning of the terms and have the best meaning in Portuguese were retained. The reliability of the consensus version was evaluated by back-translation into English by another qualified translator and assessing similarities with the original instrument.

The materials were analyzed by an experts’ committee that assessed the adequacy and clarity of the vocabulary and semantic, idiomatic, cultural, and conceptual equivalence. Changes to some items were suggested for the ease of understanding, as shown in Table 1.

After the experts’ evaluation, all the items that made up the PAC scale were verified to have a CVI = 1.00, which was considered equivalent and maintained in the pre-final version of the instrument, as shown in Table 2.

Seven caregivers participated in the pre-test: four and three in the first and second groups, respectively. Of the sample, 57.1% were women and their degree of kinship to the person cared for corresponded to sons/daughters (57.1%), sisters (14.2%), nephews (14.2%), and daughters-in-law (14,2%), in the group from 40 to 70 years old, with a mean age of 55.2 (± 4.1) years. The caregivers lived in the municipalities of São Carlos-SP (71.4%), Belo Horizonte-MG (14.2%), and Brasília-DF (14.2%). In the pre-test stage, the adapted version of the PAC scale was well accepted by family caregivers, who considered it easy to understand and fast to apply. They found no difficulty understanding the meaning and clarity of the scale items. Therefore, alterations to any questions were not necessary. The final version of the PAC scale is presented in Table 3.

**Table 1.** Evaluation of the translation and cultural adaptation of the Positive Aspects of Care scale by the experts’ committee, São Carlos, 2022

Items from the original instrument in English	Consensus version prepared by the translators	Changes proposed by the experts’ committee	Translated and adapted pre-final version
Title: Positive Aspects of Caregiving Scale	Escala de Aspectos Positivos do Cuidado		Escala de Aspectos Positivos do Cuidado
General Guidelines: Some caregivers say that, despite all the difficulties involved in giving care to a family member with memory or health problems, good things have come out of their caregiving experience too. I’m going to go over a few of the good things reported by some caregivers. I would like you to tell me how much you agree or disagree with these statements.	Alguns cuidadores dizem que, apesar de todas as dificuldades para cuidar de um familiar com problemas de memória ou de saúde, coisas boas também decorrem de sua experiência de cuidado. Vou comentar algumas dessas coisas boas relatadas por alguns cuidadores. Gostaria que você me dissesse o quanto você concorda ou discorda dessas afirmações. O cuidar...	Vou comentar algumas das coisas boas relatadas por alguns cuidadores. Gostaria que você me dissesse o quanto você concorda ou discorda dessas afirmações	Alguns cuidadores dizem que, apesar de todas as dificuldades para cuidar de um familiar com problemas de memória ou de saúde, coisas boas também decorrem de sua experiência de cuidado. Vou comentar algumas coisas boas relatadas por alguns cuidadores. Gostaria que você me dissesse o quanto você concorda ou discorda dessas afirmações. O cuidar...
**1. 1. Made me feel more useful.** 1: Disagree a lot; 2: Disagree a little; 3: Neither agree nor disagree; 4: Agree a little; 5: Agree a lot	**1. Fez com que eu me sinta mais útil.** 1: Discordo muito; 2: Discordo um pouco; 3: Nem concordo nem discordo; 4: Concordo um pouco; 5: Concordo muito.	**1. Fez com que eu me sinta mais útil.** 1: Discordo muito; 2: Discordo um pouco; 3: Nem concordo nem discordo; 4: Concordo um pouco; 5: Concordo muito.	
**2. Made me feel good about myself.** 1: Disagree a lot; 2: Disagree a little; 3: Neither agree nor disagree; 4: Agree a little; 5: Agree a lot	**2. Fez com que eu me sinta bem comigo mesmo.** 1: Discordo muito; 2: Discordo um pouco; 3: Nem concordo nem discordo; 4: Concordo um pouco; 5: Concordo muito	Mesmo(a)	**2. Fez com que eu me sinta bem comigo mesmo(a).** 1: Discordo muito; 2: Discordo um pouco; 3: Nem concordo nem discordo; 4: Concordo um pouco; 5: Concordo muito
**3. Made me feel needed.** 1: Disagree a lot; 2: Disagree a little; 3: Neither agree nor disagree; 4: Agree a little; 5: Agree a lot	**3. Fez com que eu sinta que alguém precisa de mim.** 1: Discordo muito; 2: Discordo um pouco; 3: Nem concordo nem discordo; 4: Concordo um pouco; 5: Concordo muito		**3. Fez com que eu sinta que alguém precisa de mim.** 1: Discordo muito; 2: Discordo um pouco; 3: Nem concordo nem discordo; 4: Concordo um pouco; 5: Concordo muito
**4. Made me feel appreciated.** 1: Disagree a lot; 2: Disagree a little; 3: Neither agree nor disagree; 4: Agree a little; 5: Agree a lot	**4. Fez com que eu me sinta valorizado(a)** 1: Discordo muito; 2: Discordo um pouco; 3: Nem concordo nem discordo; 4: Concordo um pouco; 5: Concordo muito		**4. Fez com que eu me sinta valorizado(a).** 1: Discordo muito; 2: Discordo um pouco; 3: Nem concordo nem discordo; 4: Concordo um pouco; 5: Concordo muito
**5. Made me feel important.** 1: Disagree a lot; 2: Disagree a little; 3: Neither agree nor disagree; 4: Agree a little; 5: Agree a lot	**5. Fez com que eu me sinta importante.** 1: Discordo muito; 2: Discordo um pouco; 3: Nem concordo nem discordo; 4: Concordo um pouco; 5: Concordo muito		**5. Fez com que eu me sinta importante.** 1: Discordo muito; 2: Discordo um pouco; 3: Nem concordo nem discordo; 4: Concordo um pouco; 5: Concordo muito
**6. Made me feel strong and confident.** 1: Disagree a lot; 2: Disagree a little; 3: Neither agree nor disagree; 4: Agree a little; 5: Agree a lot	**6. Fez com que eu me sinta forte e confiante.** 1: Discordo muito; 2: Discordo um pouco; 3: Nem concordo nem discordo; 4: Concordo um pouco; 5: Concordo muito		**6. Fez com que eu me sinta forte e confiante.** 1: Discordo muito; 2: Discordo um pouco; 3: Nem concordo nem discordo; 4: Concordo um pouco; 5: Concordo muito
**7. Enabled me to appreciate life more.** 1: Disagree a lot; 2: Disagree a little; 3: Neither agree nor disagree; 4: Agree a little; 5: Agree a lot	**7. Fez com que eu valorize mais a vida.** 1: Discordo muito; 2: Discordo um pouco; 3: Nem concordo nem discordo; 4: Concordo um pouco; 5: Concordo muito	Permitiu-me valorizar mais a vida	**7. Permitiu-me valorizar mais a vida.** 1: Discordo muito; 2: Discordo um pouco; 3: Nem concordo nem discordo; 4: Concordo um pouco; 5: Concordo muito
**8. Enabled me to develop a more positive attitude toward life.** 1: Disagree a lot; 2: Disagree a little; 3: Neither agree nor disagree; 4: Agree a little; 5: Agree a lot	**8. Permitiu-me desenvolver uma atitude mais positiva em relação à vida.** 1: Discordo muito; 2: Discordo um pouco; 3: Nem concordo nem discordo; 4: Concordo um pouco; 5: Concordo muito		**8. Permitiu-me desenvolver uma atitude mais positiva em relação à vida.** 1: Discordo muito; 2: Discordo um pouco; 3: Nem concordo nem discordo; 4: Concordo um pouco; 5: Concordo muito
**9. Strengthened my relationships with others.** 1: Disagree a lot; 2: Disagree a little; 3: Neither agree nor disagree; 4: Agree a little; 5: Agree a lot	**9. Fortaleceu minhas relações com os outros.** 1: Discordo muito; 2: Discordo um pouco; 3: Nem concordo nem discordo; 4: Concordo um pouco; 5: Concordo muito	Fortaleceu os meus relacionamentos com as outras pessoas.	**9. Fortaleceu os meus relacionamentos com as outras pessoas.** 1: Discordo muito; 2: Discordo um pouco; 3: Nem concordo nem discordo; 4: Concordo um pouco; 5: Concordo muito

**Table 2.** Percentage agreement rates among experts according to semantic and idiomatic assessments in the original and translated versions of the Positive Aspects of Care Scale. São Carlos, 2022

Items		CVI
1	Aspectos Positivos do Cuidado	1.00
2	Alguns cuidadores dizem que, apesar de todas as dificuldades para cuidar de um familiar com problemas de memória ou de saúde, coisas boas também decorrem de sua experiência de cuidado. Vou comentar algumas coisas boas relatadas por alguns cuidadores. Gostaria que você me dissesse o quanto você concorda ou discorda dessas afirmações	1.00
3	A escala de avaliação é a seguinte: 1: Discordo muito; 2: Discordo um pouco; 3: Nem concordo nem discordo; 4: Concordo um pouco; 5: Concordo muito.	1.00
4	O cuidar fez com que eu me sinta mais útil	1.00
5	O cuidar fez com que eu me sinta bem comigo mesmo(a)	1.00
6	O cuidar fez com que eu sinta que alguém precisa de mim	1.00
7	O cuidar fez com que eu me sinta valorizado(a)	1.00
8	O cuidar fez com que eu me sinta importante	1.00
9	O cuidar fez com que eu me sinta forte e confiante	1.00
10	O cuidar permitiu-me valorizar mais a vida	1.00
11	O cuidar permitiu-me desenvolver uma atitude mais positiva em relação à vida	1.00
12	O cuidar fortaleceu os meus relacionamentos com as outras pessoas	1.00

**Table 3.** Final version of the Positive Aspects of Care scale translated and culturally adapted to Brazilian Portuguese. São Carlos, 2022

Escala de Aspectos Positivos do Cuidado					
Alguns cuidadores dizem que, apesar de todas as dificuldades para cuidar de um familiar com problemas de memória ou de saúde, coisas boas também decorrem de sua experiência de cuidado. Vou comentar algumas coisas boas relatadas por alguns cuidadores. Gostaria que você me dissesse o quanto você concorda ou discorda dessas afirmações					
	Discordo muito	Discordo um pouco	Nem concordo nem discordo	Concordo um pouco	Concordo muito
1. O cuidar fez com que eu me sinta mais útil.	1	2	3	4	5
2. O cuidar fez com que eu me sinta bem comigo mesmo(a).	1	2	3	4	5
3. O cuidar fez com que eu sinta que alguém precisa de mim.	1	2	3	4	5
4. O cuidar fez com que eu me sinta valorizado(a)	1	2	3	4	5
5. O cuidar fez com que eu me sinta importante	1	2	3	4	5
6. O cuidar fez com que eu me sinta forte e confiante	1	2	3	4	5
7. O cuidar permitiu-me valorizar mais a vida	1	2	3	4	5
8. O cuidar permitiu-me desenvolver uma atitude mais positiva em relação à vida.	1	2	3	4	5
9. O cuidar fortaleceu os meus relacionamentos com as outras pessoas	1	2	3	4	5

## DISCUSSION

The current study satisfactorily implemented all the stages recommended in the literature to adapt and translate the PAC scale for caregivers of people with dementia in the Brazilian context.^15^ This process significantly contributed to the quality of the result obtained, which resulted in a Portuguese version of the instrument that is linguistically faithful to the questionnaire in its original language (English) and had its adequacy unanimously confirmed by means of content validation.

Some changes can be suggested after the translation process for the questionnaire items to be better understood by the target population. Adaptation is essential in the process because it enables cultural equivalence, as cultures differ between populations. Consequently, this affects the reliability of the results obtained by applying the instrument.^17^

For the Brazilian version of the PAC scale, a pre-test was performed with family caregivers of people with dementia belonging to different age groups, with different degrees of kinship, and of both sexes. All the participants rated the scale as easy to understand and had no difficulty answering it. Consequently, it was not necessary to implement any changes in the final version of the scale after the translation and verification processes were performed by the experts’ committee and study researchers. A systematic review of the literature on the PAC scale identified 52 studies that showed that the scale was used for multiple purposes and produced considerable evidence that it is valid and reliable, supporting the importance of understanding positive caregiving experiences.^18^

The study developed to transculturally adapt the PAC scale in Greece with caregivers of people with dementia found a result similar to the present study, as all 22 caregivers interviewed did not find any difficulties with translation of the instrument and did not have any point to note regarding the scale items.^12^ Both the translation to the Brazilian context and Greek study adopted a methodological framework involving more than one translator from English to the native language of the countries, which ensured minimizing any misinterpretations.^12^ In other countries, such as China,^19^ Japan,^14^ and Singapore,^13^ only one translator took part in each stage: translation, back-translation, and consensus on the version to be evaluated.

Notably, the Brazilian study in question included more stages to certify the translation, namely, sending the subsequent version created by the third translator to the authors for content assessment and including a fourth stage for the scale to be evaluated by a committee comprised of three judges based on the CVI. These translation and transcultural adaptation processes are necessary because of the need to assess and discuss the positive aspects of the care task. This is because implementing meaningful and efficient support strategies for this population group is possible, as there are many studies on the harms to health experienced by informal caregivers of people with dementia.^20^

Liu et al.^21^ state that it is important for health professionals and providers of community-based activities to help caregivers recognize sources of resilience that have developed across their life course and in the process of adapting to caregiving responsibility, grounded in one-on-one guidance sessions or support groups. Therefore, positive experiences related to the roles attributed to family caregivers of people with dementia include improvements in the relationship between the caregiver and the older adult, caregiver’s confidence, learning to cope with difficult circumstances, and achieving satisfaction from the care responsibilities.^22^

A notable limitation of this study is the scarcity of Brazilian studies on PAC among informal caregivers of people with dementia, which makes it difficult to deepen the discussion. Another limitation of the current study is that the data were collected using a digital platform, which precluded the participation of people with no access to the digital environment or those who were unaware of the study. However, it is important to note that this study stands out for being innovative, and that its objective is to provide an instrument about the PAC. Consequently, future studies should perform psychometric analyses of the scale to provide a reliable instrument for use in clinical practice and intervention research.

## CONCLUSION

The PAC scale was translated and culturally adapted for use by informal caregivers of people with dementia in Brazil. The findings of this study showed strong agreement among the experts in the semantic and idiomatic assessments of the original and translated versions of the scale, thus confirming that the questionnaire could be used to evaluate the positive aspects associated with caregiving in the researched sample. This study has repercussions for enabling and comparing data to global findings, in addition to providing an easy- and fast-to-apply tool for health professionals that meets the demands of caregivers of people with dementia.

## References

[1] Keating N, Eales J, Funk L, Fast J, Min J (2019). Life course trajectories of family care. Int J Care Caring.

[2] Quinn C, Toms G (2019). Influence of Positive Aspects of Dementia Caregiving on Caregivers’ Well-Being: a Systematic Review. Gerontologist.

[3] Pysklywec A, Plante M, Auger C (2020). The positive effects of caring for family carers of older adults: a scoping review. Int J Care Caring.

[4] Tarlow BJ, Wisniewski SR, Belle SH (2004). Positive aspects of caregiving: Contributions of the REACH project to the development of new measures for Alzheimer’s caregiving. Res Aging.

[5] Quinn C, Nelis SM, Martyr A (2019). Influence of positive and negative dimensions of dementia caregiving on caregiver well-being and satisfaction with life: findings from the IDEAL study. Am J Geriatr Psychiatry.

[6] Lindeza P, Rodrigues M, Costa J, Guerreiro M, Rosa MM (2020). Impact of dementia on informal care: a systematic review of family caregivers’ perceptions. BMJ Support Palliat Care.

[7] Brini S, Hodkinson A, Davies A (2022). In-home dementia caregiving is associated with greater psychological burden and poorer mental health than out-of-home caregiving: a cross-sectional study. Aging Ment Health.

[8] Tsai CF, Hwang WS, Lee JJ (2021). Predictors of caregiver burden in aged caregivers of demented older patients. BMC Geriatr.

[9] Pinyopornpanish M, Pinyopornpanish K, Soontornpun A (2021). Perceived stress and depressive symptoms not neuropsychiatric symptoms predict caregiver burden in Alzheimer’s disease: a cross-sectional study. BMC Geriatric.

[10] Gonçalves-Pereira M, Carmo I, da Silva JA (2010). Caregiving experiences and knowledge about dementia in Portuguese clinical outpatient settings. Int Psychogeriatr.

[11] Roth DL, Dilworth-Anderson P, Huang J, Gross AL, Gitlin LN (2015). Positive Aspects of Family Caregiving for Dementia: Differential Item Functioning by Race. J Gerontol B Psychol Sci Soc Sci.

[12] Tsatali M, Egkiazarova M, Toumpalidou M (2022). Greek adaptation of the Positive Aspects of Caregiving (PAC) Scale in dementia caregivers. Clin Gerontol.

[13] Siow JYM, Chan A, Østbye T, Cheng GH, Malhotra R (2017). Validity and reliability of the Positive Aspects of Caregiving (PAC) Scale and development of its shorter version (S-PAC) among family caregivers of older adults. Gerontologist.

[14] Furukawa H, Greiner C (2021). Reliability and validation of the Positive Aspects of Caregiving scale among Japanese caregivers of people with dementia. Int J Nurs Sci.

[15] Beaton DE, Bombardier C, Guillemin F, Ferraz MB (2000). Guidelines for the process of cross-cultural adaptation of self-report measures. Spine.

[16] Lynn MR (1986). Determination and quantification of content validity. Nurs Res.

[17] Cordeiro TLR, de Souza JM (2021). Tradução, validação e adaptação transcultural de instrumento para ensino de cricotireoidostomia por punção. Espac Saúde.

[18] Lee Y, Li L (2022). Evaluating the Positive Experience of Caregiving: A Systematic Review of the Positive Aspects of Caregiving Scale. Gerontologist.

[19] Lou VW, Lau BH, Cheung KS (2015). Positive aspects of caregiving (PAC): scale validation among Chinese dementia caregivers (CG). Arch Gerontol Geriatr.

[20] Sakanashi S, Fujita K, Konno R (2021). Empowerment among family caregivers of community-dwelling people with dementia in Japan: a qualitative research study. J Nurs Res.

[21] Liu J, Lou Y, Wu B, Mui ACYS (2021). “I’ve been always strong to conquer any suffering:” challenges and resilience of Chinese American dementia caregivers in a life course perspective. Aging Ment Health.

[22] Kyei-Arthur F, Codjoe SNA, Badasu DM (2022). Exploring positive experiences of primary and secondary caregivers of older persons in resource-limited urban settings in Accra, Ghana. PLoS One.

